# Respiratory Tract Infections in Inflammatory Bowel Disease Patients Taking Vedolizumab: A Systematic Review and Meta-Analysis of Randomized Controlled Trials

**DOI:** 10.3389/fphar.2020.585732

**Published:** 2021-01-22

**Authors:** Irene Marafini, Edoardo Troncone, Irene Rocchetti, Giovanni Monteleone

**Affiliations:** ^1^ Chair of Gastroenterology, Department of Systems Medicine, University of Rome Tor Vergata, Rome, Italy; ^2^ Statistical Office, Superior Council of Judiciary, Rome, Italy

**Keywords:** crohn’ disease, ulcerative colitis, coronavirus, pneumonia, integrin, α4β7

## Abstract

The ongoing COVID-19 pandemic has raised concerns about the risk of SARS-CoV-2 infection in patients with Crohn’s disease (CD) and patients with ulcerative colitis (UC) taking immunosuppressants or biologics. We conducted a systematic review and meta-analysis to assess the risk of respiratory infections in patients with inflammatory bowel disease (IBD) treated with vedolizumab. We searched PubMed, EMBASE and Scopus to identify randomized controlled trials (RCT) comparing vedolizumab to placebo in patients with IBD. Outcomes were the rate of respiratory tract infections (RTI), upper respiratory tract infections (URTI) and lower respiratory tract infections (LRTI) among patients receiving vedolizumab as compared with placebo. Pooled rates were reported as Odds Ratios (OR) with 95% Confidence Interval (CI). Eight RCT involving 3,287 patients (1873 CD and 1415 UC) were analyzed; 2,493 patients received vedolizumab and 794 received placebo. The rates of RTI and URTI were statistically higher in vedolizumab-treated patients compared to placebo [OR = 1.63; 95% CI (1.07–2.49); OR = 1.64 95% CI (1.07–2.53) respectively]. UC patients, but not CD patients, receiving vedolizumab had a higher risk to develop RTI and URTI [OR = 1.98; 95% CI (1.41–2.77); OR = 2.02; 95% CI (1.42–2.87)] compared to placebo-treated patients. The number of LRTI was small in both treatment groups. Data confirm the good safety profile of vedolizumab even though RTI were more frequent in patients receiving vedolizumab and the risk of URTIs was significantly higher in patients with UC.

## Introduction

Vedolizumab is a humanized monoclonal antibody approved for the treatment of active Crohn's Disease (CD) and ulcerative Colitis (UC). Vedolizumab binds to the α4β7 integrin thus preventing interaction of such an integrin with the adhesion molecule mucosal vascular addressin cell adhesion molecule 1 (MAdCAM-1) expressed on the vascular endothelium. This determines inhibition of the trafficking of activated T and B lymphocytes to the gut (Soler et al., 2009; Marafini et al., 2014; Zundler et al., 2019). Phase-3, double-blind, placebo-controlled studies of vedolizumab in UC (GEMINI 1) and CD (GEMINI 2) showed that the drug is effective as induction and maintenance therapy for the treatment of both disorders (Feagan et al., 2013; Sandborn et al., 2013). Effectiveness of the drug was confirmed by many real-world observational studies (Chaparro et al., 2018; Christensen et al., 2018; Lenti et al., 2018; Plevris et al., 2019; Scarozza et al., 2020). It was also reported that patients with less severe disease and naïve to tumor necrosis factor (TNF) blockers are more likely to respond to vedolizumab (Dulai et al., 2016; Amiot et al., 2017; Favale et al., 2019; Hupe et al., 2020; Meserve and Dulai, 2020). Overall, the above studies showed also the drug is well-tolerated with an acceptable safety profile (Colombel et al., 2017). In particular, when data from GEMINI 1, GEMINI 2, and a long-term safety study in UC and CD were pooled, it was shown that the incidence of upper respiratory tract infections (URTI) was numerically higher in patients receiving vedolizumab compared with those receiving placebo, although this difference was not statistically significant (Feagan et al., 2018). However, the GEMINI two study showed that, during the maintenance phase, the incidences of infections and of serious infections, including respiratory tract infections (RTI), were higher with vedolizumab than with placebo (Sandborn et al., 2013). Today, this latter finding could be particularly relevant because of the outbreak of coronavirus disease 2019 (COVID-19) caused by the novel severe acute respiratory syndrome coronavirus 2 (SARS-CoV-2) (Munster et al., 2020; Zhu et al., 2020). The clinical spectrum of SARS‐CoV‐2 ranges from asymptomatic or mild respiratory disease to pneumonia with respiratory distress syndrome, which can in some cases lead to a fatal outcome (Guan et al., 2020). Although, inflammatory bowel disease (IBD) patients seem to have no increased risk to be infected with SARS‐CoV‐2 (Bezzio et al., 2020; Monteleone and Ardizzone, 2020), physicians need evidences to decide whether to continue or hold specific treatments during this pandemic. To determine the risk of RTIs with vedolizumab in patients with IBD, we performed a systematic review and meta-analysis of randomized clinical trials (RCTs).

## Methods

The methods of our analysis and inclusion criteria were based on Preferred Reporting Items for Systematic Reviews and Meta-Analyses (PRISMA) recommendations (Shamseer et al., 2015). Our protocol was registered with PROSPERO on May 2020, after searching for ongoing systematic reviews (protocol number: CRD42020185760).

### Data Source and Search Strategy

A comprehensive literature search was performed using PubMed, Embase and Scopus (from inception up to May 2020) to identify RCTs regarding the safety of vedolizumab in IBD. The electronic search was supplemented by manual screening of the studies. We identified studies using the following medical subject headings (MeSH) and keywords including: “vedolizumab”, “alpha4beta7”, “α4β7”, “anti-integrin” “and ‘inflammatory bowel disease’. The Medline search strategy was (“vedolizumab” [Supplementary Concept] OR “vedolizumab” [All Fields]) OR alpha4beta7 [All Fields] OR anti-integrin [All Fields] OR alpha4beta7 [All Fields] AND (“inflammatory bowel diseases” [MeSH Terms] OR (“inflammatory” [All Fields] AND “bowel” [All Fields] AND “diseases” [All Fields]) OR “inflammatory bowel diseases” [All Fields] OR (“inflammatory” [All Fields] AND “bowel” [All Fields] AND “disease” [All Fields]) OR “inflammatory bowel disease” [All Fields]).

### Outcome Assessment

The primary outcome measure of interest was the rate of RTIs among patients receiving vedolizumab compared with placebo. Secondary outcomes included rate of upper respiratory tract infections (URTI) and lower respiratory tract infections (LRTI) and sub-analysis according to disease type (CD or UC).

### Inclusion and Exclusion Criteria

As for inclusion criteria, we considered RCTs reporting the incidence of adverse events (AE). We included only article in English and conducted in adult patients with IBD. We determined the number of each AE by article. RCTs conducted in healthy subjects receiving vedolizumab were excluded from the analysis. RCTs without an arm receiving placebo were excluded from the final analysis. Therefore, we excluded from the analysis head-to-head studies comparing either vedolizumab with other active drugs or different routes of vedolizumab administration. Subgroup analysis were also performed separately in CD and UC.

### Selection Process

Two authors independently searched each of the potential trials to determine whether they were eligible for inclusion. Uncertainties or disagreements were resolved with a consensus among the authors. Full reports were obtained for all titles meeting the inclusion criteria. Full reports were retrieved even in case of any uncertainty. Then, the review authors decided whether to include the retrieved studies by screening the full reports.

### Data Extraction

Using standardized forms, two reviewers (IM and ET) extracted data independently. Discrepancies were resolved by discussion. An arbitrator (GM) resolved unresolved disagreements. The following data were extracted from each of the included studies: year of publication, study design, study population, patients characteristics (age, gender, IBD type), study duration (follow-up) and treatment duration, number of RTI [nasopharyngitis, URTI, influenza-like syndrome, lower respiratory tract infections (LRTI), and pneumonia for vedolizumab and placebo-treated patients]. In the final analysis, RTI were divided in two groups: URTI (including also nasopharyngitis and influenza-like syndrome) and LRTI (including also pneumonia).

We included in the vedolizumab group all the patients who received at least one dose of vedolizumab during the study period. Therefore, patients who completed the induction with vedolizumab and then were assigned to maintenance therapy with placebo were included in the vedolizumab group. The placebo group included patients who were never exposed to vedolizumab throughout the whole study duration.

### Statistical Analysis

The global effect size was calculated taking into account the effects size of the single study analyses. Odds ratio (OR) was used to estimate total and single-study effect size. According to the existence of heterogeneity among the different studies, verified by applying the Q Cochran test (*x2*), random effect models (DerSimonian Laird method) were used to calculate pooled ratios for all the included studies and for the CD patients. The Mantel-Haenszel fixed effect model was used to calculate pooled OR for UC patients, given that the Q test in this case was not statistically significant. The statistical software R has been used for all the analysis made. Forests plots were provided to compare the OR of the single studies with respect to the combined Effect Size and to show graphically the confidence intervals and the significance of the estimates.

## Results

### Study Characteristics

**FIGURE 1:**
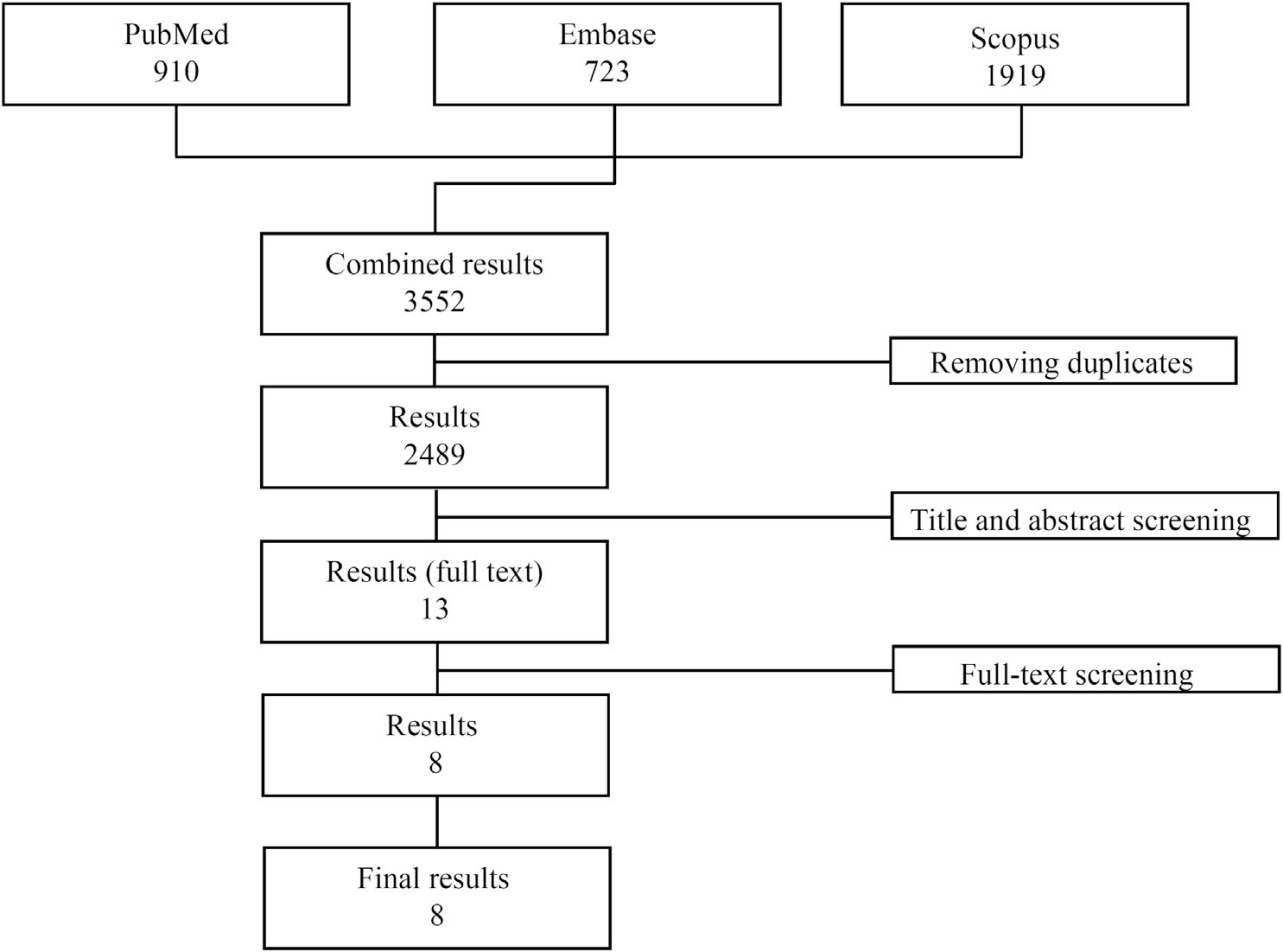
Study flow-chart.

A total of 3,552 publications were initially identified (PubMed: 910, Embase: 723, Scopus: 1919). After removal of all the duplicates, 2,489 studies were evaluated and after evaluation of the abstracts, only 13 studies were considered for the full text revision. Of these, five studies were excluded: 1 because it was not a randomized controlled trial, 2 because the study population included healthy subjects only and 2 because they did not have a placebo group. Eight studies matching the inclusion/exclusion criteria were finally included in the analysis ((Feagan et al., 2013); (Sandborn et al., 2013); (Feagan et al., 2005; Feagan et al., 2008; Parikh et al., 2012; Sands et al., 2014; Motoya et al., 2019; Watanabe et al., 2020)) (Figure 1). All studies were multicentre, randomized placebo-controlled clinical trials published between 2005 and 2020 and conducted across Canada, United States, Europe, Russia and Japan. Overall, the mean treatment duration was 30.25 weeks (range 4–54 weeks), while the mean follow-up was 37.9 weeks (range 8–60 weeks). In total, 3,287 patients (1873 CD and 1415 UC) were randomized: 2,493 exposed to at least one dose of vedolizumab and 794 receiving placebo. The mean age range across the studies was 35.7–43.8 for vedolizumab-treated patients and 32.6–44 for placebo-treated patients. One thousand-two hundred-eighty-six out of 2,493 vedolizumab-treated patients and 425 out of 794 placebo-treated patients were male.

### Respiratory Tract Infections are More Frequent in Patients Receiving Vedolizumab

**FIGURE 2:**
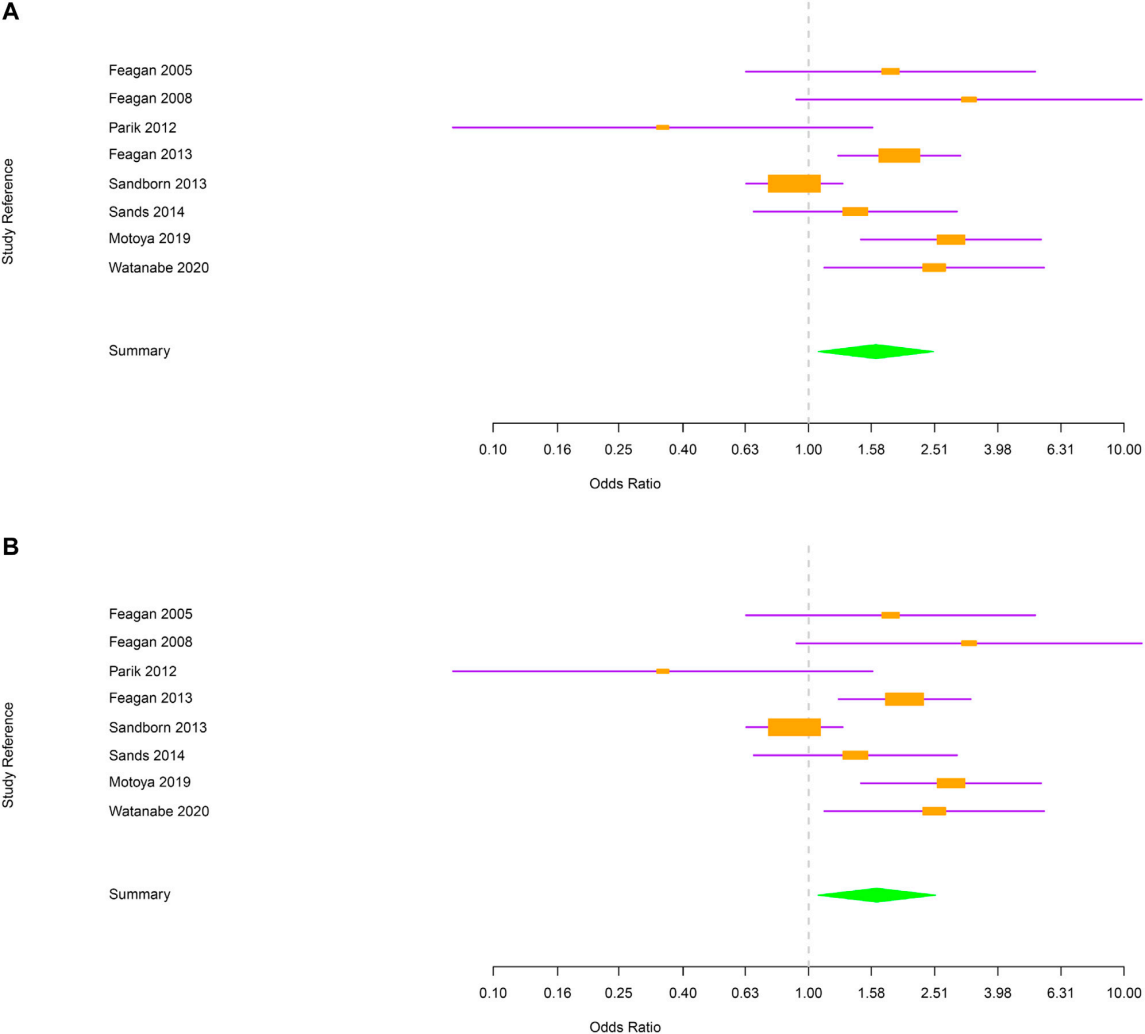
**(A)** Forest plot for the rate of respiratory tract infections in inflammatory bowel disease patients. **(B)** Forest plot for the rate of upper respiratory tract infections in inflammatory bowel disease patients.

Among vedolizumab-treated patients, 762 cases of RTI were reported: 731 were URTI and 31 were LRTI. Placebo-treated patients experienced 138 RTI: 133 URTI and 5 LRTI. URTI, including nasopharyngitis, were the most frequent RTI in both treatment groups (Table 1). The number of RTI was significantly higher in the group taking vedolizumab as compared to placebo [OR = 1.63; 95% CI (1.07–2.49); Figure 2A]. When the analysis was restricted to URTI, the positive association between vedolizumab and RTI was confirmed [OR = 1.64 95%CI (1.07–2.53); Figure 2B). The number or LRTI was small in both treatment groups, and despite LRTI were numerically higher in patients taking vedolizumab compared to placebo (31 vs 5), no statistical analysis could be made.

**TABLE 1:** Number of patients enrolled in the indicated randomized, placebo-controlled clinical studies and number of respiratory tract infections in patients treated with either vedolizumab or placebo.

References	Patients vedolizumab	Patients placebo	Type of IBD	RTI vedolizumab	URTI vedolizumab	LRTI vedolizumab	RTI placebo	URTI placebo	LRTI placebo
Feagan et al. (2005)	118	63	UC	16	16	0	5	5	0
Feagan et al. (2008)	127	58	CD	19	19	0	3	3	0
Parikh et al. (2012)	37	9	UC	8	8	0	4	4	0
Feagan et al. (2013)	746	149	UC	224	193	31	27	22	5
Sandborn et al. (2013)	967	148	CD	381	381	0	62	62	0
Sands et al. (2014)	209	207	CD	18	18	0	13	13	0
Motoya et al. (2019)	210	82	UC	73	73	0	13	13	0
Watanabe et al. (2020)	79	78	CD	23	23	0	11	11	0

IBD, inflammatory bowel disease; CD, Crohn’s disease; UC, ulcerative colitis; RTI, respiratory tract infections; URTI, upper respiratory tract infections; LRTI, lower respiratory tract infections.

**FIGURE 3:**
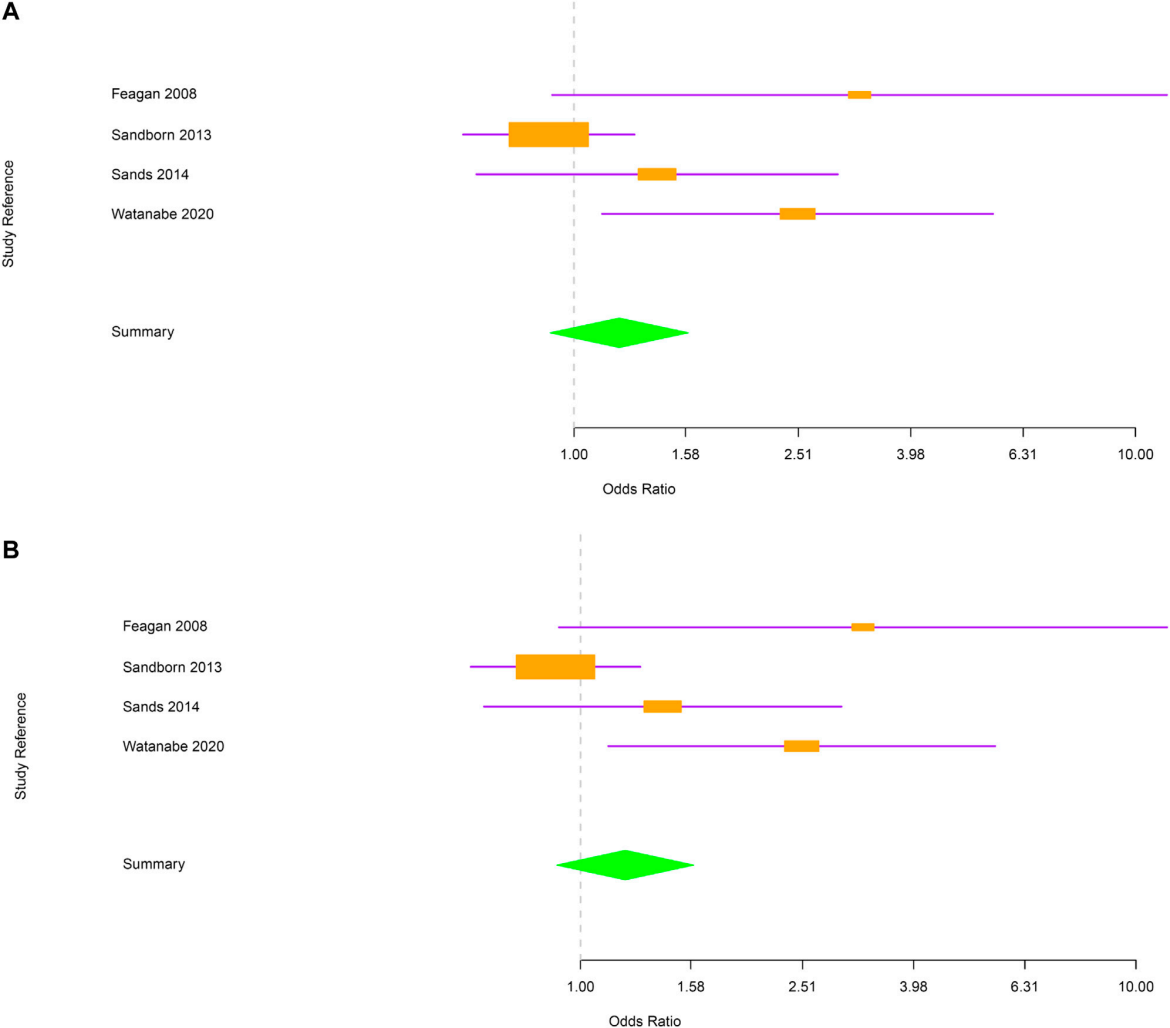
**(A)** Forest plot for the rate of respiratory tract infections in Crohn’s disease patients. **(B)** Forest plot for the rate of upper respiratory tract infections in Crohn’s disease patients.

**FIGURE 4:**
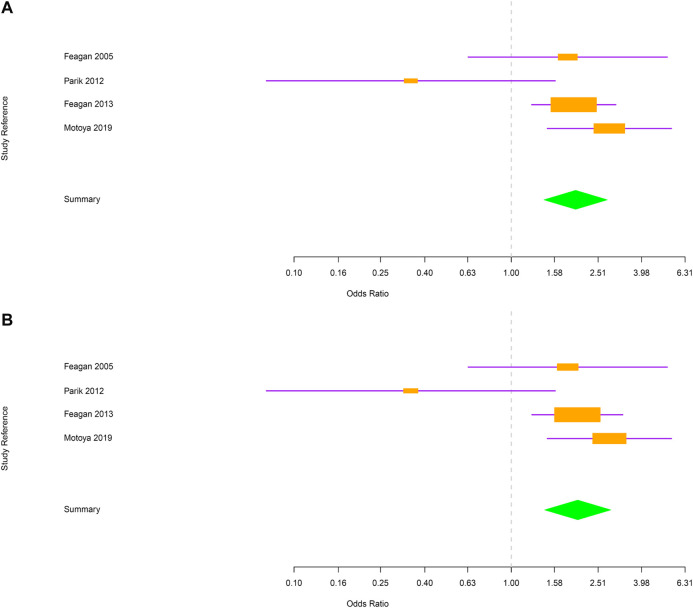
**(A)** Forest plot for the rate of respiratory tract infections in ulcerative colitis patients. **(B)** Forest plot for the rate of upper respiratory tract infections in ulcerative colitis patients.

Next, evaluations were performed separately for CD and UC. In CD, the risk of RTI and URTI was not statistically different between vedolizumab and placebo-treated patients [OR = 1.2; 95% CI (0.91–1.6) and OR = 1.2; 95% CI (0.91–1.6) respectively; Figure 3A,B]. In UC group, patients treated with vedolizumab had a higher risk to develop RTI or URTI than those receiving placebo [OR = 1.98; 95% CI (1.41–2.77); OR = 2.02; 95% CI (1.42–2.87), respectively; Figure 4A,B].

## Discussion

The SARS-CoV-2 infection, which appeared in China in December 2019 and rapidly spread throughout the world, has forced clinicians to carefully evaluate the risk that IBD patients might have to develop COVID-19, as some drugs used to halt the IBD-associated tissue damaging inflammatory response may have an impact on infectious rates (Downey, 2016) and are known to increase incidence of RTI, including pneumonia (Kennedy et al., 2020; Sarzi-Puttini et al., 2020). At the same time, IBD by itself carries on an increased risk of pneumonia. We believe it is relevant to make a distinction between URTI and LRTI, as URTI are generally self-limiting diseases, while the latter are more difficult to treat and usually require the use of antibiotics (Godman et al., 2020).

In a retrospective cohort study, Long and colleagues demonstrated that the risk of developing pneumonia was approximately 50% higher for IBD patients than for the general population (Long et al., 2013). In a United States hospitalization database, Ananthakrishnan and colleagues showed that more than one fourth of all-related hospitalisations were attributable to infections, which caused excess mortality risk. Patients with pneumonia had the highest excess mortality risks compared with patients without infection-related hospitalization (Ananthakrishnan and McGinley, 2013). In these studies, the risk of infection among IBD patients was further enhanced by anti-TNF agents. Consistently, data emerging from a large prospective registry, indicate that the rates of serious infection rates, including RTI, were reported to be higher in the group of infliximab-treated CD patients than in patients receiving other non-biological therapies (Lichtenstein et al., 2018). In this context, it is however noteworthy that the risk of infection in patients receiving TNF blockers could be somewhat influenced by the underlying disease. Indeed, no increased risk of RTI was found in psoriatic patients treated with anti-TNF (Syed et al., 2020a). The risk of RTI following treatment with other biologics in psoriasis still has to be determined (Syed et al., 2020b).

The present meta-analysis was undertaken to determine whether patients exposed to vedolizumab have increased risk of RTI. Through analysis of the safety profile of vedolizumab documented in placebo-controlled RCTs involving 2,493 patients treated with vedolizumab and 794 treated with placebo, we showed that the number of RTI was significantly higher in the group of vedolizumab-treated patients than in the placebo group and this was evident for the URTI but not LRTI. Surprisingly, the risk of URTI among vedolizumab treated-patients was increased in UC but not in CD. Factors accounting for such a discrepancy remain unknown but it is conceivable that differences in the presence of confounders (e.g. smoking, concomitant use of steroids, immunesuppressors, or TNF blockers) among the two IBD populations can account for the increased risk of URTI in the vedolizumab-treated UC patients. However, we did not have access to the individual data of the patients enrolled in the RCTs and, therefore, we cannot ascertain the impact of such factors in the greater frequency of URTI in vedolizumab-treated UC patients.

The increased frequency of URTI in vedolizumab-treated UC patients might be explained by the expression of MAdCAM-1 in the oropharynx (Prados et al., 2018) and, therefore, vedolizumab treatment could block migration of T cells involved in the host defense against pathogens (e.g., CD8^+^ T cells) toward the upper respiratory mucosa.

Our results are conflicting with those published by Feagan et al., who demonstrated that in the two GEMINI RCTs (n = 1731 patients) there was no statistical difference in terms of URTI between patients receiving vedolizumab and those receiving placebo (Feagan et al., 2018). However, in the same analysis, the authors reported that the incidence of URTIs was numerically higher in patients receiving vedolizumab compared with those receiving placebo. A possibility is that such a discrepancy reflects differences in the number of RCTs and patients analyzed. In fact, in contrast to the Feagan’s analysis, we extended our evaluation to eight RCTs. Another possibility is that in the GEMINI studies, safety outcomes were evaluated separately for induction and maintenance therapy and for patients receiving vedolizumab at different intervals, while in our analysis all the vedolizumab-treated patients were grouped together. Moreover, in the assessment of AE, both GEMINI studies included in the placebo group patients receiving vedolizumab in the induction phase and then assigned to placebo in the maintenance phase. In contrast, these patients were included in the vedolizumab group in our study as we cannot exclude the possibility that adverse events documented during the maintenance phase could depend on a carry-over effect of the drug. Consistently, our placebo group included exclusively those patients who never received vedolizumab treatment. Our data differ also, in part, from those published by Colombel and colleagues, who documented no increased risk of infections, including RTI, in patients exposed to vedolizumab. Notably, such an analysis included six double-blind or open-label trials and did not take into consideration other RCTs evaluating the safety profile of vedolizumab, which were included in the present meta-analysis (Colombel et al., 2017).

In our study, the incidence of LRTI was low in both treatment groups, even though a trend toward higher rates was observed in vedolizumab-treated patients. Interestingly, no pneumonia was evident from our study, while Feagan and colleagues reported that 11 vedolizumab-treated and two placebo-treated patients developed pneumonia, a finding that was not reported in the original GEMINI trial (Feagan et al., 2013; Sandborn et al., 2013). This reflects the fact that some RCTs indicate only AE occurring in more than 2–5% of the patients and some cases of bronchopneumonia can be classified as “any serious infection”.

Comforting results on the use of other biologics are present in the latest literature. For example, in a large cohort study, adult COVID-19 patients with recent anti-TNF exposure did not have increased rates of hospitalization or mortality compared with patients without recent anti-TNF exposure (Yousaf et al., 2020).

We are aware that our study has some limitations. In RCTs, the screening procedure excludes the patients with potential risk factors for RTI (e.g,. cardio-vascular and respiratory comorbidities, older age). Thus, the carefully selected IBD patient population in RCTs does not reflect exactly the more refractory or complicated IBD patient populations treated in clinical practice. Moreover, the safety data considered in our analysis referred to studies with short-term follow-up, so we could not assess the long-term effect of vedolizumab on RTIs because the RCTs in our study had placebo-controlled periods of no more than 54 weeks. As mentioned above, additional confounders for RTI could not be evaluated due to the lack of individual patient data, even though in RCTs researchers randomize usually the patients taking into account all the known confounders.

We excluded non-controlled, real-world experiences from our analysis as they may not take into consideration minor adverse events (e.g., URTI) occurring during treatments and generally lack a placebo group, which makes difficult to ascertain the real risk of RTI.

In conclusion, our study confirms the good safety profile of vedolizumab, even though RTI were more frequent in patients receiving vedolizumab and the risk of URTIs was significantly higher in patients with UC. These findings help clinicians to ensure patients about the safety of vedolizumab especially during the ongoing COVID-19 pandemic, even though a case-by-case decision about continuing/withdrawing major disease modifying treatments is always advised.

## Author Contributions

IM and ET performed the research, collected and analyzed the data, designed the research study and wrote the paper; IR analyzed the data; GM designed the research study and wrote the paper. All authors approved the final.

## Conflict of Interest

The authors declare that the research was conducted in the absence of any commercial or financial relationships that could be construed as a potential conflict of interest.
